# In Memoriam

**Published:** 1879-04

**Authors:** 


### IN MEMORIAM.

Mrs. Emma L. Day, wife of Dr. D. C. Day, died at Bolivar, Hardeman county, Tenn , on the 24th of March. We tender our heartfelt sympathy to our bereaved brother.

---

On the 16th inst., the State Medical Association of Georgia meets in Rome, Ga. We hope that the meeting will be pleasant, beneficial and harmonious, and that the mutual interchange of ideas and information may be abundant and profitable.

